# Cannabinoid CB2 Receptor Gene and Environmental Interaction in the Development of Psychiatric Disorders

**DOI:** 10.3390/molecules23081836

**Published:** 2018-07-24

**Authors:** Hiroki Ishiguro, Yasue Horiuchi, Koichi Tabata, Qing-Rong Liu, Tadao Arinami, Emmanuel S. Onaivi

**Affiliations:** 1 Department of Neuropsychiatry and Clinical Ethics, Graduate School of Medical Science, University of Yamanashi, Chuo, Yamanashi 409-3898, Japan; tabata-ymn@umin.org; 2 Department of Psychiatry and Behavioral Sciences, Tokyo Metropolitan Institute of Medical Science, Setagaya, Tokyo 156-8506, Japan; horiuchi-ys@igakuken.or.jp; 3 National Institute on Aging-IRP, NIH, Baltimore, MD 21224, USA; qingrong.liu@nih.gov; 4 Department of Medical Genetics, Graduate School of Comprehensive Human Sciences, University of Tsukuba, Tsukuba, Ibaraki 305-8575, Japan; tadao.arinami@gmail.com; 5 Department of Biology, William Paterson University, Wayne, NJ 07470, USA

**Keywords:** endocannabinoid, cannabinoid CB2 receptor, *Cnr2*, depression, anxiety, chronic mild stress, neuro-immune signaling, HPA axis

## Abstract

CB2 cannabinoid receptor (CB2R) gene is associated with depression. We investigated the gene-environment interaction between CB2R function and diverse stressors. First, anxiety-like behavior during chronic-mild-stress (CMS) was evaluated in C57BL/6JJmsSlc mice following treatment with CB2R agonist JWH015 or inverse-agonist AM630. Second, locomotor activity and anxiety-like behavior were measured following exposure to an immune poly I:C stressor. Gene expressions of HPA axis related molecules, *Fkbp5*, *Nr3c1* and *Crf* and pro-inflammatory cytokine *Il-1b*, as well as *Bdnf* as a key neurotrophin that supports neuron health, function, and synaptic plasticity, were determined in hippocampus of *Cnr2* knockout mice, as indicators of stressful environment. CMS-induced anxiety-like behavior was enhanced by AM630 and reduced by JWH015 and fluvoxamine. Poly I:C reduced locomotor activity and increased anxiety-like behavior, and these effects were pronounced in the heterozygote than in the wild type mice. *Fkbp5* and *Nr3c1* expression were lower in the *Cnr2* heterozygotes than in the wild type mice with Poly I:C treatment. These findings indicate that interaction between CB2R gene and stressors increases the risk of depression-like behaviors that may be linked with neuro-immune crosstalk. Further studies in human subjects are necessary to determine the role of CB2R and environmental interaction in the development of depression.

## 1. Introduction

While most studies focus on the CB1 receptor (CB1R), recognition of the importance of understanding the roles of the cannabinoid CB2 receptor (CB2R) in the central nervous system in neuropsychiatric disorders has been increasing [1]. Reduced function of the CB2R is associated with several psychiatric disorders including schizophrenia, major depression, and substance abuse [2,3,4], but these genetic findings need to be validated by behavioral experiments with animal models of psychiatric disorders. The polymorphisms in the CB2R gene (*CNR2*) are frequent, and are commonly observed in human population [2] associated with psychiatric disorders [5]. In addition, our studies on the association of human *CNR2* gene polymorphisms suggested that these polymorphisms are a common factor in some psychiatric disorders. We have previously demonstrated that *Cnr2* gene expression was downregulated in the brains of the mice subjected to chronic-mild-stress (CMS) and also in mice that developed alcohol preference [3].

The endocannabinoid system appears to contribute to stress response, including activation of the hypothalamic-pituitary-adrenal (HPA) axis and increases in anxiety-related behavior during stress [6]. As stress responses involves the HPA axis and affect the onset and maintenance of stress-related disorders including depression [7], genes that play a role in the HPA-axis regulation may likely contribute to stress-related disorders. Within the canonical HPA axis, we examined the *FKBP5*, *CRF* and *NR3C1* in this study. FKBP5, a co-chaperone of hsp90, is responsible for regulation of GR sensitivity to cortisol [8,9]. FKBP5 has been reported to be one of the biomarkers for stress-response and for effects of antidepressant action in patients with depression [8,10,11,12]. The *NR3C1*, encoding GR, is known as a major component of HPA axis against stress, and have been reported to be one of stress biomarkers with responses to psychosocial stress following the Trier Social Stress Test (TSST) [13]. The polymorphisms, methylation and expression of the *NR3C1* gene have been indicated to be associated with depression and especially related to stress [14,15,16,17,18,19]. The *CRF* gene, that codes corticotropin-releasing factor, contributes to stress-related depressiveness [20]. We hypothesized that if the endocannabinoid system is involved in HPA axis, then the expression of *Fkbp5*, *Crf* and *Nr3c1* genes may be associated with changes in CB2R function in mice. Neuro-immune crosstalk mediated by stressors is an important target of study in psychiatric disorders. Previous studies have indicated changes in expression of cytokines in psychiatric disorders [21,22,23,24,25,26,27,28,29]. Differences in proinflammatory cytokines produced by monocytes between patients with major depressive disorder and healthy controls [30]. Exposure to chronic restraint stress exacerbated allodynia and depressive-like behavior along with increase in Interleukin 1β (IL-1β) gene expression [31]. GR antagonist reduced depression-like behaviors induced by Il-1b administration in rodent model [32]. Brain-derived neurotrophic factor (BDNF) gene expression in blood was found to be negatively associated with depression score (HAMD) [33], and Bdnf/trkB signaling was reduced in the hippocampus of genetic model of vulnerability to stress-induced depression [34]. We therefore examined *Il1b* and *Bdnf* gene expressions that have been implicated as biomarkers for depression.

The endocannabinoid system is associated with stress-induced psychiatric disorders. Chronic-mild-stress (CMS) has been developed on the basis of stress-diathesis hypothesis of depression [35]. The viral mimic polyinosinic-polycytidilic acid, Poly I:C is a synthetic double-stranded RNA which activates the Toll-like receptor 3 (TLR3) pathway, and which is used in animal model of depression [36]. Therefore, the effects of CMS and Poly I:C treatments were analyzed in this study to simulate stressors such as emotional and immune stressors.

The aim of this study was to determine the role of CB2Rs and environmental factors on behavioral characteristics of mouse models of depression using two stressors, and to evaluate neuro-inflammatory and HPA axis biomarkers in the stress models. This study is a first report of our research focusing on the gene-environment interaction between CB2R and stressors in the mature mouse.

## 2. Results

All statistical results for both behavioral and gene expression analysis are shown in Table 1.

### 2.1. Behavioral Analysis

We examined whether two important components of depressive behavior, anxiety and hypoactivity using the stressed animals. In the Zero maze test, there were no differences in the percent time spent in open section between the wild type and the heterozygote *Cnr2* KO mice where all the mice were naïve (Table 1).

**Figure 1:**
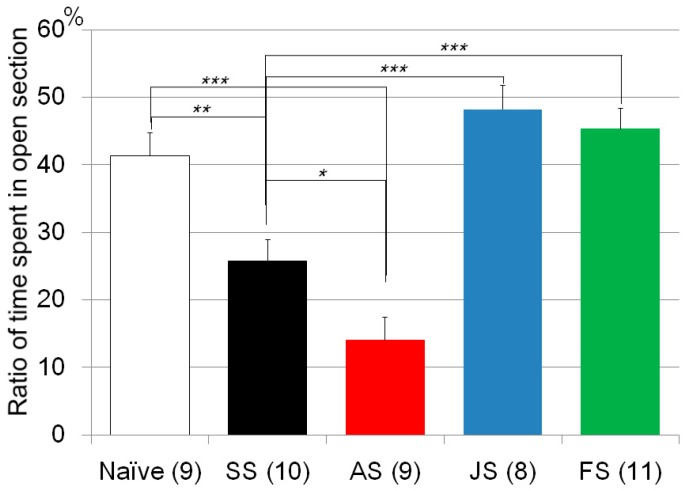
Cannabinoid CB2R ligands regulate CMS-induced anxiety-like behavior. The vertical axis shows %time spent in open section in Zero maze. Naïve is non-stressed control, and others are CMS treated mice. White bar is %time of the Naïve (non-stressed) mice, black bar is %time of the stressed with saline treatment mice (SS), red bar are %time of the stressed with AM630 treatment mice (AS), blue bar is %time of the stressed with JWH015 treatment mice (JS), and green line bar is %time of the stressed with fluvoxamine treatment mice (FS). Number of subjects for each group is indicated in parenthesis. Significant difference between the groups: * *p* = 0.015, ** *p* = 0.0016, *** *p* < 1 × 10^−3^.

As stress model, CMS enhanced anxiety-like behavior of the JJmsSlc mice in Zero maze compared with that of the CMS-naïve mice. CMS differentially altered the anxiety-like behavior of mice treated with saline, CB2R agonist, inverse agonist and fluvoxamine (Figure 1 and Table 1). After CMS, AM630 treatment significantly augmented the anxiety-like behavior compared with the saline treated mice. In contrast, JWH015 reduced the anxiety-like behavior induced by CMS, which was similar to the effect of antidepressant fluvoxamine (Figure 1 and Table 1).

**Figure 2:**
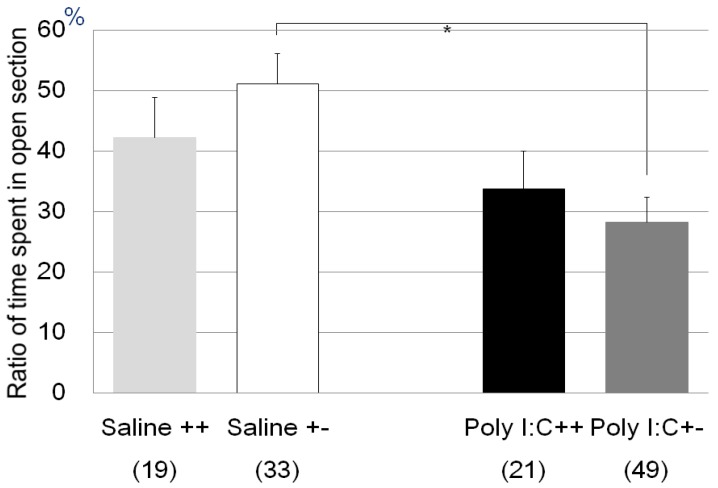
Anxiety-like behavior following immune stress induced by Poly I:C injection in Zero maze test. The vertical axis shows %time spent in the open section of the Zero maze. Light gray bar is %time of the wild type saline treated mice, white bar is %time of the heterozygote saline treated mice, black bar is %time of the wild type Poly I:C treated mice, and dark gray bar is %time of the heterozygotes Poly I:C treated mice. The higher %time spent in the open section indicates reduced anxiety-like behavior of the mice. * Significant difference was found between treatments in heterozygote knockout mice as shown (*p* = 0.003), while no difference was found in wild type mice.

Treatment with Poly I:C reduced the percent time spent in the open section of the Zero maze, indicating an anxiety-like behavior that was enhanced in the heterozygote *Cnr2* knockout mice than in the wild type mice (Figure 2 and Table 1).

**Figure 3:**
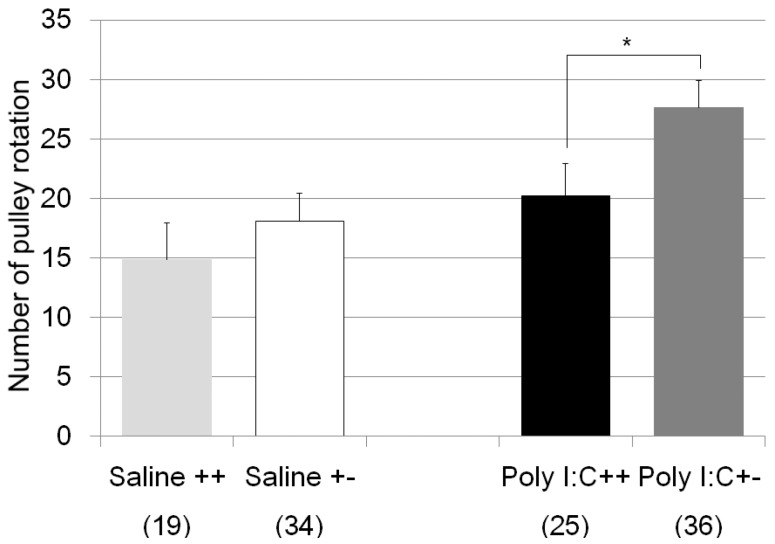
Anxiety-like behavior of mice in the rotated pulley model induced by Poly I:C injection. Mice that display anxiety-like behavior rotated the pulley faster in order to crawl up from water. The vertical axis shows number of pulley rotation. Light gray bar indicates the wild type saline treated mice, white bar represents the heterozygote saline treated mice, black bar represents the wild type Poly I:C treated mice, and dark gray bar represents the heterozygote Poly I:C treated mice. Number of subjects for each group is shown in parenthesis. There was * Significant difference between the genotypes when the mice were treated with Poly I:C (*p* = 0.03).

**Figure 4:**
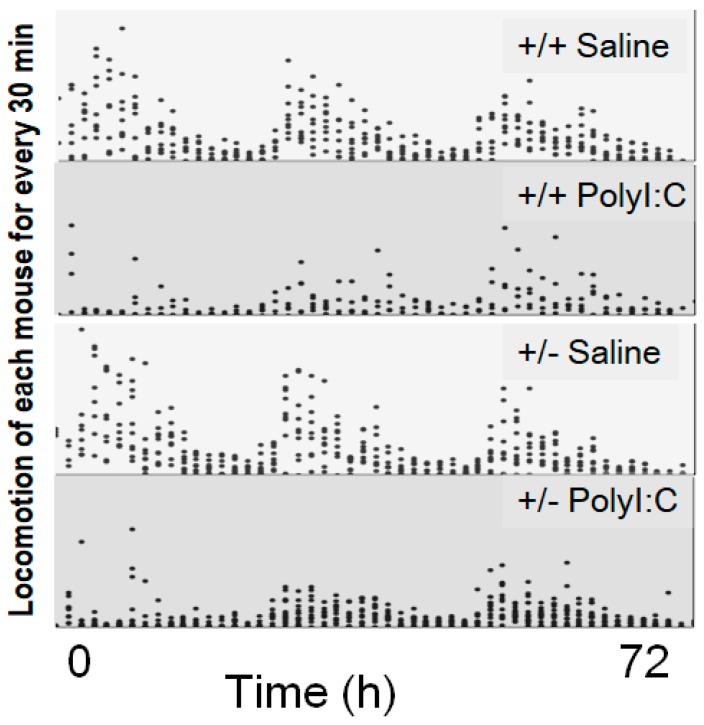
Locomotor activity of the *Cnr2* heterozygote KO mice three days after Poly I:C injection. Locomotor activity of each mouse every 30 min for 72 h is plotted as a dot for the wild type and the heterozygote *Cnr2* KO mice. The light gray fields shows the locomotor activity of saline treated mice, while dark gray field show that of Poly I:C treated mice. ANOVA analysis showed significant difference (*p* < 0.0001), especially for the effect of Poly I:C treatment (*p* < 0.0001), but not for the genotypes (*p* = 0.35) or the combination of Poly I:C treatment and genotypes (*p* = 0.17). However, post-hoc analysis showed a small but insignificant difference in locomotor activity between the genotypes when the mice were injected with Poly I:C (*p* (single sided) = 0.06).

Similar anxiety-like behavior were obtained with the number of rotation of the pulley in the test box, with the pulley placed half-sunk into water (Figure 3 and Table 1). It is also important to evaluate a possibility that stressed mice show hypolocomotion. In the locomotor activity test there was no significant difference but there was a tendency for the heterozygote *Cnr2* KO mice to reduce their locomotor activities in their home cages compared to the wild type mice during 72 h after the Poly I:C injection (Figure 4 and Table 1). Poly I:C, that is known known to induce immune stress, reduced the locomotor activity of mice and provoked anxiety-like behavior, which was apparent in the *Cnr2* heterozygotes than in the wild type mice. This provides additional evidence that components of the endocannabinoid system, including Cb2r are implicated in regulating the stress response and involved in neuro-immune signaling underlying depression

### 2.2. Gene Expression

**Figure 5:**
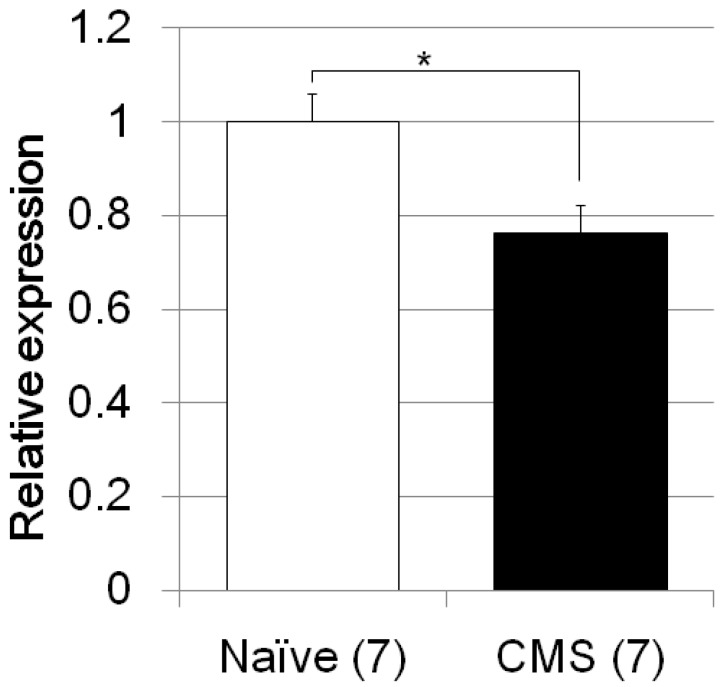
*Fkbp5* gene expression in the hippocampus of the C57B/6JJmsSlc mice is altered by CMS. The vertical axis shows the relative *Fkbp5* expression level in the hippocampus of CMS treated C57BL/6JJmsSlc mice in comparison to that of naïve mice. Black bar indicates the relative *Fkbp5* expression of the stressed mice in comparison to that of naïve (non-stressed) mice indicated in white bar. Number of subjects for each group is shown in parenthesis. There was * Significant reduction of the expression of *Fkbp5* in the hippocampus in mice with CMS (*p* = 0.013).

It has not been clearly understood, neuro-immune and HPA axis function seemed to work in vulnerability to psychiatric disorders, and either in development or in result of the disorders. Thus some molecules had been indicated as biomarker for stress and/or depression. Our study could support the biomarker related to HPA axis. CMS reduced the *Fkbp5* gene expression significantly in the hippocampus of C57B/JJmsSlc mice (Figure 5 and Table 1).

**Figure 6:**
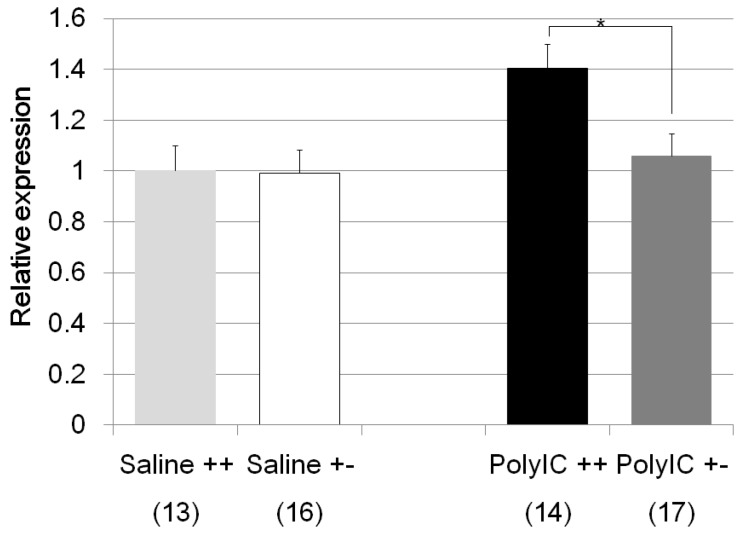
Analysis of the *Fkbp5* gene expression in the hippocampus after Poly I:C treatment. The vertical axis shows the relative *Fkbp5* expression level after Poly I:C or saline treatments for each genotype group (in comparison to saline treated wild type mice). Light gray bar indicates the wild type saline treated mice, white bar represents the heterozygote saline treated mice, black bar represents the wild type Poly I:C treated mice, and dark gray bar represents the heterozygote Poly I:C treated mice. Number of subjects in each group is shown in parenthesis. * Significant difference between the genotypes when the mice were treated with Poly I:C (*p* = 0.01).

Poly I:C treatment significantly reduced the expression of *Fkbp5* in the *Cnr2* heterozygote KO mice than in the wild type controls, while the saline treatment had no effect in the expression of *Fkbp5* in mice (Figure 6 and Table 1).

**Figure 7:**
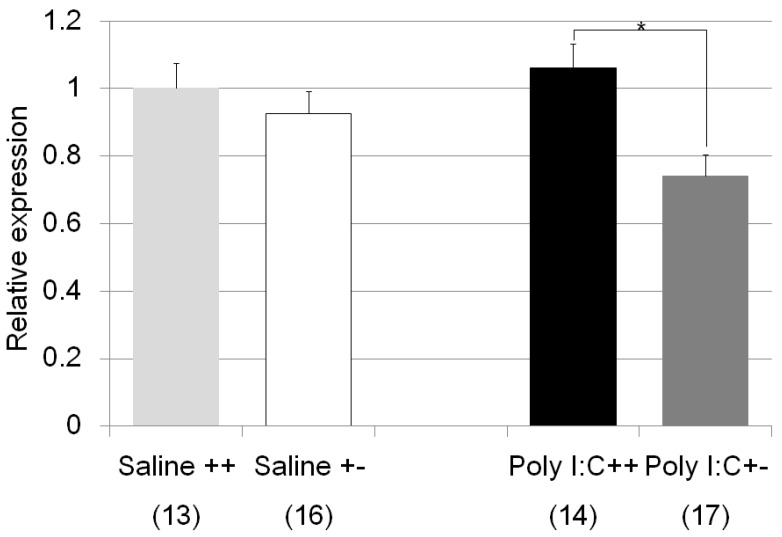
Analysis of the *Nr3c1* gene expression in the hippocampus of *Cnr2* KO mice treated with Poly I:C. The vertical axis shows the relative *Nr3c1* expression level after Poly I:C treatment for each genotype group (in comparison to saline treated wild type mice). Light gray bar indicates the wild type saline treated mice, white bar represents the heterozygote saline treated mice, black bar represents the wild type Poly I:C treated mice, and dark gray bar represents the heterozygote Poly I:C treated mice. Number of subjects in each group is shown in parenthesis. * Significant difference between the genotypes when the mice were treated with Poly I:C (*p* = 0.007).

There was a significant reduction in the expression of *Nr3c1* gene in the heterozygotes than in the wild type mice after Poly I:C treatment, and no differences was found in the expression of *Nr3c1* in the saline-treated mice between the genotypes. (Figure 7 and Table 1).

**Figure 8:**
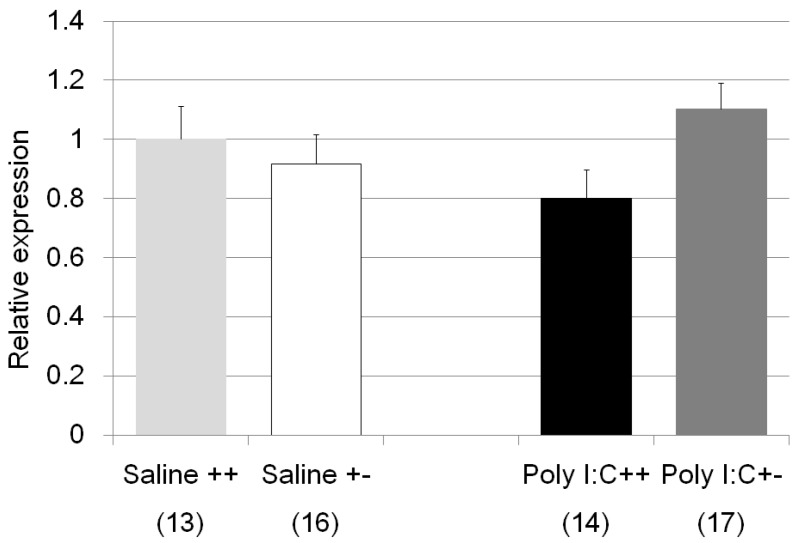
Analysis *Il1b* gene expressions in the hippocampus after poly I:C stress. The vertical axis shows the relative *Il1b* expression level after Poly I:C treatment for each genotype group (in comparison to saline treated wild type mice). Light gray bar indicates the wild type saline treated mice, white bar represents the heterozygote saline treated mice, black bar represents the wild type Poly I:C treated mice, and dark gray bar represents the heterozygote Poly I:C treated mice. The numbers of the subjects are shown in parenthesis. While two-way ANOVA indicated possible involvement of Il1b in genotype and Poly I:C treatment, post-hoc analysis with Tukey test did not show any significant differences between the groups.

However, there were no difference in the expression of *Crf* gene in the hippocampus between the genotype of the mice and between saline and Poly I:C treatments. Regard of neuro-inflammation signaling, although the expression of interleukin *Il1b* gene seemed to be more in the heterozygotes than in the wild type mice with Poly I:C treatment, Tukey test did not show any difference between each group. (Figure 8 and Table 1). The stressors used in this study reduced *Fkbp5* and *Nr3c1* gene expressions in hippocampus associated with emotionality and reward pathways and reveals a role for CB2Rs in the mouse models of depression.

## 3. Discussion

There is increasing evidence that cannabinoid CB2 receptors (CB2Rs) are involved in brain function and in neuropsychiatric disorders. The present study indicates that genetically induced low function of the CB2Rs increases susceptibility to depression when combined with emotional (CMS) and immune (poly I:C) stressors. This is consistent with our previous reports that low CB2R function induces depression-like symptoms in the mouse model and alcohol addiction in human population [3]. FKBP5/Fkbp5 is one of the biomarkers for posttraumatic stress disorder (PTSD): The *FKBP5* gene expression was reported to be low but elevated during cognitive behavioral therapy (CBT) in PTSD patients [37,38]. Consistent with the PTSD study, the results from our experiments using a mouse model demonstrated a reduction of the *Fkbp5* gene expression after CMS. However, another study reported that CMS increased Fkbp5 protein expression, which was not observed in rats with adrenalectomy [39]. The reason for the difference is not clear, but may be due to species’ difference, and difference in measurements or CMS procedures utilized. It this study, reduced *Fkbp5* gene expression in hippocampus is indicated to be a biomarker for the developed anxiety-like behavior developed by CMS (C vs. SS in Figure 1 and Figure 5). Reduced *Fkbp5* and *Nr3c1* gene expressions in the brain of the *Cnr2* heterozygote knockout mice in comparison to wild type mice indicated the developed anxiety-like behavior by Poly I:C stress shown in rotated pulley test (Figure 3, Figure 6 and Figure 7), which are also useful as biomarkers, although the anxiety behavior shown in Zero maze test seemed not to be very distinct parallel to the alteration of *Fkbp5* expression in mice brain (Figure 2, Figure 6 and Figure 7).

There is also growing interest in role of neuro-immune crosstalk and signaling in the development of psychiatric disorders [40]. CB2Rs are linked to the immune system function in the periphery and CNS, and are involved in neural progenitor proliferation in the hippocampus [41,42]. In this study, Poly I:C, known to produce immune stress, decreased the locomotor activity of mice and increased their anxiety-like behavior, which was apparent in the *Cnr2* heterozygotes than in the wild type mice. This provides additional evidence for the involvement of endocannabinoid system in neuro-immune reaction of the central nervous system underlying depression.

The current study examined environmental risk factors in the mature brain in the mouse as model of depression. However human patients may show vulnerability to certain types of stressors in developing psychiatric conditions like depression and schizophrenia that may be age dependent. For example, maternal immune activation (MIA) and adolescent cannabinoid exposure (ACE) have both been identified as major environmental risk factors for schizophrenia [43]. Cannabis use in adolescents was reported to be one of the risk factors for schizophrenia [44] and recent work with animal models demonstrated that adolescence would be a vulnerable period for developing schizophrenia [45]. Although influence of MIA on schizophrenia development was not clear in the general population, more studies of the interaction of MIA and the endocannabinoid system are needed. This is because neonatal rats experiencing maternal deprivation showed anomalous behaviors in adulthood which are also observed in schizophrenic patients [46]. Furthermore, similar mechanisms could occur in development of stress-related disorders, including depression, anxiety disorders and alcoholism [47,48], which could be observed also in “depressive-like” animal model [49]. Animal studies indicated that early maternal deprivation is associated with increase in the number of degenerating hippocampal neurons and astrocytes and increased corticosterone and implicating the endocannabinoid system as 2-AG levels increased in the hippocampus [50,51]. Long-term changes in monoamines and Bdnf, as well as in HPA-axis function, were induced by early maternal deprivation [52,53]. Further studies are needed for age-dependent distress on development of stress-related disorders including depression.

In the present study, Poly I:C treatment differentially altered the expression of *Fkbp5* and *Nr3c1* in brains of mice stressed with Poly I:C either between genotypes of mice or in contrast to saline treatment. The alteration of IL-1β level, as well as that of BDNF [33,54], has been observed in major depressive patients, according to systematic Review and Meta-Analysis [28]. In the present study, Poly I:C treatment differentially altered the expression of interleukin *Il1b* gene in the striatum between the *Cnr2* heterozygotes and the wild type mice. It remains unclear whether the specific cytokine affects the neural system to develop depression uniquely. Each stressor affects different cytokines, which causes different neuro-immune reaction induced endophenotypes that lead to different psychiatric disturbances. Further studies on the roles of cytokines in brain pathology are now focused on revealing the risks of combining specific stressors and vulnerable ages in the development of depression, schizophrenia, and other psychiatric diseases. Transcriptome analysis of cytokine expression in animals as well as in human patients is also necessary to understand the specific roles of CB2Rs and interaction with HPA axis and neuro-immune signaling in psychiatric disorders.

The present study has two limitations; first, it was performed in animal models for depression and needs to be tested in human subjects. Second, we examined whether adult brain stressors would lead to the development of psychiatric symptoms. While acute stressors may cause depression in human adults, future studies of possible interactions between hypomorphic CB2R function and the impact of stressors are required to characterize the developmental roles of the CB2Rs in psychiatric disorders.

## 4. Materials and Methods

The experimental design and the tested animals in this study were summarized in Table 2. All study participants provided informed consent, and the study design was approved by the Research Ethics Committee of Faculty of Medicine, University of Yamanashi and the Research Ethics Committee of University of Tsukuba, Japan.

### 4.1. Subjects

Mice were housed under 12 h light/12 h dark cycle and with food and water *ad libitum*. Two strains of mice were used in this study. The first group, *Cnr2* knockout mice were provided by Prof. Buckley [55] and the heterozygous mating pairs were obtained from the same litter and were bred by heterozygote-heterozygote breeding strategies so that the average genetic backgrounds of the knockout mice were identical to produce the cohorts used in this study. In order to represent human subjects with genetically low CB2 receptor function, we did not use homozygote *Cnr2* knockout mice. The second group, C57BL/6JJmsSLC mice (Japan SLC, Inc., Shizuoka, Japan) were treated with CB2R ligands and used for CMS test. Mice were group housed. The study was approved by the Institutional Animal Care and Use Committee at University of Yamanashi and at University of Tsukuba.

### 4.2. Behavioral Analysis

Behavioral changes were analyzed following the application of two stressors to determine the functional relationship of the stressors with CB2Rs. The application of CMS and immune stressor using Poly I:C to the animals were analyzed for possible roles in anxiety-like and depression-like behaviors. All behavioral tests were performed during light phase.

### 4.3. Anxiety-Like Behavioral Test

The CMS treatment was implemented as follows: mice were housed separately from other mice in home cages with either no-food, no-water, tilted and no-bedding tips, wet bedding, or strobe-light with heat, which were applied twice daily for 2 weeks. C57BL/6JJmsSlc male mice (age, 8–10 weeks: weight, 20–25 g: n = 8–11 for each group) (Japan SLC, Inc.) were divided into five groups. Group one (n = 9) was a control without CMS treatment. Groups two through five were given CMS, with each group (n = 8–11) concurrently injected intraperitoneally with either saline, CB2 inverse agonist AM630 (3 mg/kg), CB2 agonist JWH015 (20 mg/kg), or fluvoxamine (30 mg/kg). Their trend of anxiety was measured by elevated Zero maze (San Diego Instruments Inc., San Diego, CA, USA), in order to evaluate a simple influence from chronic mild stress without additional stresses, such as forced swimming, we chose Zero maze for suitable experiment in this study [56]. Briefly, each mouse is free to walk for 5 min on circular elevated passage that is divided into 4 equal portions. Two opposite sections have walls on both sides (enclosed) while the other two opposite sections have no walls (open). The time spent in enclosed section was measured and regarded as anxiety-like behavior. Next, the effect of immune stressor with poly I:C was analyzed. *Cnr2* knockout and naive male mice (age, 8–10 weeks: weight, 20–25 g: n = 19–49 for each group) were pre-administered intraperitoneally (ip) with Poly I:C (6 mg/kg) were used for anxiety-like behavioral test in the elevated Zero maze and in a new model to access anxiety-like behavior. Briefly, this new anxiety test was performed in the test box where a pulley was placed half-sunk into water, because commonly used forced-swim test itself has given additional inconsiderable stress onto the test mice [56]. When each mouse to be tested was put beside the pulley, we counted how many times he rotated the pulley in order to try to get out of the water during five minutes test period. The test was initiated 30 s after the mice were placed in the water.

### 4.4. Locomotor Activity Test

*Cnr2* heterozygote knockout male mice (age, 8–10 weeks: weight, 20–25 g: 5 Poly I:C treated and 9 saline treated wild type mice, and 15 Poly I:C treated and 10 saline treated heterozygote mice) administered intraperitoneally with Poly I:C (6 mg/kg) were monitored individually for locomotor activity in the home cages. Locomotor activity was measured by an activity monitoring system (ACTIMO-100; Shinfactory, Fukuoka, Japan) using ACTIMO-DATA software [57] Data were collected every 30 min for 72 h.

### 4.5. Gene Expression Analysis

*Fkbp5* gene expression, as biomarker of depression and stress-related disorders (Figure 5), was analyzed in the hippocampus from the mice subjected to CMS and immunologically stressed (Poly I:C). To confirm the role of CB2R in HPA axis, nuclear receptor subfamily 3 group C member 1 (*Nr3c1*) and corticotropin releasing hormone (*Crf*) gene expressions were analyzed in immunologically stressed (Poly I:C) mice. Interleukin(Il) 1β and *Bdnf* gene expressions were analyzed for stress induced neuro-immune reaction in the mice. Total RNA was extracted from the brain tissue with RNeasy Mini Kit (QIAGEN, Hombrechtikon, Switzerland). cDNA was synthesized with Revertra Ace (Toyobo, Tokyo, Japan) and oligo dT primer from RNA. Expression of target genes was analyzed with an ABI PRISM 7900 HT Sequence Detection System (Applied Biosystems, Foster City, CA, USA) with the TaqMan gene expression assays for *Fkbp5* (Mm00487406_m1), *Nr3c1* (Mm00433832_m1), *Crf* (Mm001293920_m1) and *Il1b* (Mm00434228_m1). Rodent GAPDH Control Reagents (#4308313, Applied Biosystems) was used to normalize the target gene expression. TaqMan assay was performed in 10 μL reaction volumes. Measurement of threshold cycle (Ct) was the average of four replicates.

### 4.6. Statistics

Percent time-spent in Zero maze, and rotation number in another anxiety test were analyzed by two-way ANOVA, followed by post-hoc analysis for comparison of the locomotion at each time-period using the Tukey’s test. Locomotor activity was compared between groups of mice with different treatments by two-way ANOVA. The difference of expression level of *Fkbp5* gene in JJmsSlc mice with between naïve and CMS was analyzed by one-way ANOVA. The difference of expression level of *Fkbp5* gene in JJmsSlc mice with between naïve and CMS was analyzed by one-way ANOVA. The difference of expression level of each other gene between knockout mice genotypes in either treatment was analyzed by two-way ANOVA, followed by post-hoc analysis using the Tukey’s test. ANOVA and Tukey’s tests were carried out by using JMP software version 13 (SAS Institute, Japan).

## 5. Conclusions

In conclusion, reduced CB2R function in the endocannabinoid system plays a role in the occurrence of anxiety-like and depression-like related behaviors, during exposure to environmental stressors. This study provides evidence that cannabinoid CB2 receptors could be a potential target, and CB2R agonists may be therapeutic ligands for psychiatric disorders like depression.

## Figures and Tables

**Table 1:** Result of statistical analyses of the behavioral and gene expression data. The results of the statistical analysis for each experiment of the behavioral and gene expression data are shown. The numbers in parenthesis show the corresponding figures. The results by ANOVA are shown in general and in each effect. Post-hoc analysis with Tukey tests was performed where appropriate. Differences were considered significant at the a priori level of *p* < 0.05.

Behavioral Test	Treatment/Strain	Statics ANOVA	Noted by Post-Hoc Analysis
***Behavior***			
Zero maze test	Saline/*Cnr2* ko mice (no Figure)	F1,34 = 0.48, *p* = 0.49	
Zero maze test	Poly I:C/*Cnr2* ko mice (Figure 1)	F3,118 = 4.4, *p* = 0.0055	Poly I:C vs. saline treated heterozygote mice; *p* = 0.0033
		genotype F = 0.09, *p* = 0.76	
		treatment F = 7.7, *p* = 0.006	
		genotype × treatment F = 1.6, *p* = 0.20	
pulley rotate test	Poly I:C/*Cnr2* ko mice (Figure 2)	F3,110 = 4.8, *p* = 0.0034	Poly I:C treated wildtype mice vs. heterozygote mice; *p* = 0.019
		genotype F = 4.2, *p* = 0.04	
		treatment F = 8.2, *p* = 0.005	
		genotype × treatment F = 0.6, *p* = 0.42	
Zero maze test	CMS/C57B/JJmsSlc mice (Figure 3)	F4, 46 = 19.0, *p* < 0.0001	CMS treated vs. naïve mice; *p* = 0.0016
			AM630 vs. Saline treated mice with CMS; *p* = 0.015
			JWH015 vs. Saline treated mice with CMS; *p* < 1 × 10^−3^
			Fluvoxamine vs. Saline treated mice with CMS; *p* < 1 × 10^−3^
			JWH015 vs. Fluvoxamine treated mice with CMS; *n.s.*
Locomotion test	Poly I:C/*Cnr2* ko mice (Figure 4)	t(1827) = 1.54, *p* (single sided) = 0.06	
***Gene expression***			
*Fkbp5*	CMS/C57B/JJmsSlc mice (Figure 5)	F1, 12 = 8.4, *p* = 0.0134	
*Fkbp5*	Poly I:C/*Cnr2* ko mice (Figure 6)	F3,58 = 4.6, *p* = 0.008	Poly I:C treated wildtype mice vs. heterozygote mice; *p* = 0.048
		genotype F = 2.6, *p* = 0.01	Poly I:C treated vs. Saline treated wildtype mice; *p* = 0.025
		treatment F = −1.9, *p* = 0.06	Poly I:C treated wildtype mice vs. Saline treated heterozygote mice; *p* = 0.013
		genotype × treatment F = −1.7, *p* = 0.08	
*Nr3c1*	Poly I:C/*Cnr2* ko mice (Figure 7)	F3,59 = 4.4, *p* = 0.008	Poly I:C treated wildtype mice vs. heterozygote mice; *p* = 0.007
		genotype F = 8.4, *p* = 0.005	
		treatment F = 0.83, *p* = 0.37	
		genotype x treatment F = 3.2, *p* = 0.078	
*Il1b*	Poly I:C/*Cnr2* ko mice (Figure 8)	F3,58 = 1.8, *p* = 0.17	*n.s.* (Poly I:C treated wildtype mice vs. heterozygote mice; *p* = 0.14)
		genotype F = 0.0004, *p* = 0.98	
		treatment F = 0.98, *p* = 0.33	
		genotype × treatment F = 4.05, *p* = 0.049	
*Bdnf*	Poly I:C/*Cnr2* ko mice (no figure)	F3,59 = 0.15, *p* = 0.93	n.s.
*Crf*	Poly I:C/*Cnr2* ko mice (no figure)	F3,59 = 0.54, *p* = 0.66	n.s.
		genotype F = 0.49, *p* = 0.63	
		treatment F = 0.54, *p* = 0.59	
		genotype × treatment F = 0.97, *p* = 0.34	

**Table 2:** The experimental design and the tested animals. The stressors, chemicals administered to mice and behavioral tests used in the study are shown. The numbers in parenthesis in experimental treatment section show number of mice used.

Stressors	Experimental Treatment	Behavioral Tests	Gene Expression Analysis
Physical/Emotional (Chronic mild stress)	Control (10) AM630 (9) JWH015 (8) Fluvoxamine (11) Saline (10)	1. Zero maze	*Fkbp5* Control (7) vs. Saline (7)
Immune	Poly I:C (20) Saline (19)	1. Locomotion in home cage	*Fkbp5*, *Il1b*, *Nr3c1*, *Bdnf*, *Crf* Poly I:C (31) vs. Saline (29)
	Poly I:C (70) Saline (52)	2. Zero maze	
	Poly I:C (61) Saline (53)	3. Pulley rotating test (originally developed test)	

## References

[1] Onaivi E.S., Ishiguro H., Gu S., Liu Q.R. (2012). CNS effects of CB2 cannabinoid receptors: Beyond neuro-immuno-cannabinoid activity. J. Psychopharmacol..

[2] Ishiguro H., Horiuchi Y., Ishikawa M., Koga M., Imai K., Suzuki Y., Morikawa M., Inada T., Watanabe Y., Takahashi M. (2010). Brain cannabinoid CB2 receptor in schizophrenia. Biol. Psychiatry.

[3] Ishiguro H., Iwasaki S., Teasenfitz L., Higuchi S., Horiuchi Y., Saito T., Arinami T., Onaivi E.S. (2007). Involvement of cannabinoid CB2 receptor in alcohol preference in mice and alcoholism in humans. Pharmacogenomics J..

[4] Onaivi E.S., Ishiguro H., Gong J.P., Patel S., Meozzi P.A., Myers L., Perchuk A., Mora Z., Tagliaferro P.A., Gardner E. (2008). Functional expression of brain neuronal CB2 cannabinoid receptors are involved in the effects of drugs of abuse and in depression. Ann. N. Y. Acad. Sci..

[5] Ishiguro H., Onaivi E.S. (2017). Beyond the Kraepelinian Dichotomy of Schizophrenia and Bipolar Disorder. J. Schizophr. Res..

[6] Morena M., Patel S., Bains J.S., Hill M.N. (2016). Neurobiological Interactions between Stress and the Endocannabinoid System. Neuropsychopharmacology.

[7] Gerritsen L., Milaneschi Y., Vinkers C.H., van Hemert A.M., van Velzen L., Schmaal L., Penninx B.W. (2017). HPA Axis Genes, and Their Interaction with Childhood Maltreatment, are Related to Cortisol Levels and Stress-Related Phenotypes. Neuropsychopharmacology.

[8] Binder E.B. (2009). The role of FKBP5, a co-chaperone of the glucocorticoid receptor in the pathogenesis and therapy of affective and anxiety disorders. Psychoneuroendocrinology.

[9] Denny W.B., Valentine D.L., Reynolds P.D., Smith D.F., Scammell J.G. (2000). Squirrel monkey immunophilin FKBP51 is a potent inhibitor of glucocorticoid receptor binding. Endocrinology.

[10] Gassen N.C., Hartmann J., Schmidt M.V., Rein T. (2015). FKBP5/FKBP51 enhances autophagy to synergize with antidepressant action. Autophagy.

[11] Fabbri C., Hosak L., Mossner R., Giegling I., Mandelli L., Bellivier F., Claes S., Collier D.A., Corrales A., Delisi L.E. (2016). Consensus paper of the WFSBP Task Force on Genetics: Genetics, epigenetics and gene expression markers of major depressive disorder and antidepressant response. World J. Biol. Psychiatry.

[12] Young D.A., Inslicht S.S., Metzler T.J., Neylan T.C., Ross J.A. (2018). The effects of early trauma and the FKBP5 gene on PTSD and the HPA axis in a clinical sample of Gulf War veterans. Psychiatry Res..

[13] Kumsta R., Entringer S., Koper J.W., van Rossum E.F., Hellhammer D.H., Wust S. (2007). Sex specific associations between common glucocorticoid receptor gene variants and hypothalamus-pituitary-adrenal axis responses to psychosocial stress. Biol. Psychiatry.

[14] Palma-Gudiel H., Cordova-Palomera A., Tornador C., Falcon C., Bargallo N., Deco G., Fananas L. (2018). Increased methylation at an unexplored glucocorticoid responsive element within exon 1D of NR3C1 gene is related to anxious-depressive disorders and decreased hippocampal connectivity. Eur. Neuropsychopharmacol..

[15] Kang H.J., Bae K.Y., Kim S.W., Shin I.S., Kim H.R., Shin M.G., Yoon J.S., Kim J.M. (2018). Longitudinal associations between glucocorticoid receptor methylation and late-life depression. Prog. Neuropsychopharmacol. Biol. Psychiatry.

[16] Sarubin N., Hilbert S., Naumann F., Zill P., Wimmer A.M., Nothdurfter C., Rupprecht R., Baghai T.C., Buhner M., Schule C. (2017). The sex-dependent role of the glucocorticoid receptor in depression: Variations in the NR3C1 gene are associated with major depressive disorder in women but not in men. Eur. Arch. Psychiatry Clin. Neurosci..

[17] Park S., Hong J.P., Lee J.K., Park Y.M., Park Y., Jeon J., Ahn M.H., Yoon S.C. (2016). Associations between the neuron-specific glucocorticoid receptor (NR3C1) Bcl-1 polymorphisms and suicide in cancer patients within the first year of diagnosis. Behav. Brain Funct..

[18] Yin H., Galfalvy H., Pantazatos S.P., Huang Y.Y., Rosoklija G.B., Dwork A.J., Burke A., Arango V., Oquendo M.A., Mann J.J. (2016). Glucocorticoid Receptor-Related Genes: Genotype and Brain Gene Expression Relationships to Suicide and Major Depressive Disorder. Depress. Anxiety.

[19] Schatzberg A.F., Keller J., Tennakoon L., Lembke A., Williams G., Kraemer F.B., Sarginson J.E., Lazzeroni L.C., Murphy G.M. (2014). HPA axis genetic variation, cortisol and psychosis in major depression. Mol. Psychiatry.

[20] Chen L., Li S., Cai J., Wei T.J., Liu L.Y., Zhao H.Y., Liu B.H., Jing H.B., Jin Z.R., Liu M. (2018). Activation of CRF/CRFR1 signaling in the basolateral nucleus of the amygdala contributes to chronic forced swim-induced depressive-like behaviors in rats. Behav. Brain Res..

[21] Kageyama Y., Kasahara T., Kato M., Sakai S., Deguchi Y., Tani M., Kuroda K., Hattori K., Yoshida S., Goto Y. (2017). The relationship between circulating mitochondrial DNA and inflammatory cytokines in patients with major depression. J. Affect. Disord..

[22] Misiak B., Stanczykiewicz B., Kotowicz K., Rybakowski J.K., Samochowiec J., Frydecka D. (2017). Cytokines and C-reactive protein alterations with respect to cognitive impairment in schizophrenia and bipolar disorder: A systematic review. Schizophr. Res..

[23] Wu J.Q., Chen D.C., Tan Y.L., Tan S.P., Xiu M.H., Wang Z.R., Yang F.D., Soares J.C., Zhang X.Y. (2016). Altered interleukin-18 levels are associated with cognitive impairment in chronic schizophrenia. J. Psychiatr. Res..

[24] Al-Hakeim H.K., Al-Rammahi D.A., Al-Dujaili A.H. (2015). IL-6, IL-18, sIL-2R, and TNFalpha proinflammatory markers in depression and schizophrenia patients who are free of overt inflammation. J. Affect. Disord..

[25] Shirts B.H., Wood J., Yolken R.H., Nimgaonkar V.L. (2008). Comprehensive evaluation of positional candidates in the IL-18 pathway reveals suggestive associations with schizophrenia and herpes virus seropositivity. Am. J. Med. Genet. B Neuropsychiatr. Genet..

[26] Xiu M.H., Chen D.C., Wang D., Zhang K., Dong A., Tang W., Zhang F., Liu L.J., Liu J.H., Liu H.B. (2012). Elevated interleukin-18 serum levels in chronic schizophrenia: Association with psychopathology. J. Psychiatr. Res..

[27] Zhang X.Y., Tang W., Xiu M.H., Chen D.C., Yang F.D., Tan Y.L., Wang Z.R., Zhang F., Liu J., Liu L. (2013). Interleukin 18 and cognitive impairment in first episode and drug naive schizophrenia versus healthy controls. Brain Behav. Immun..

[28] Kohler C.A., Freitas T.H., Stubbs B., Maes M., Solmi M., Veronese N., de Andrade N.Q., Morris G., Fernandes B.S., Brunoni A.R. (2018). Peripheral Alterations in Cytokine and Chemokine Levels after Antidepressant Drug Treatment for Major Depressive Disorder: Systematic Review and Meta-Analysis. Mol. Neurobiol..

[29] Ellul P., Boyer L., Groc L., Leboyer M., Fond G. (2016). Interleukin-1 beta-targeted treatment strategies in inflammatory depression: Toward personalized care. Acta Psychiatr. Scand..

[30] Zhang H.X., Xu Y.Q., Li Y.Y., Lu M.F., Shi S.X., Ji J.L., Wang L.W. (2018). Difference in proinflammatory cytokines produced by monocytes between patients with major depressive disorder and healthy controls. J. Affect. Disord..

[31] Norman G.J., Karelina K., Zhang N., Walton J.C., Morris J.S., Devries A.C. (2010). Stress and IL-1beta contribute to the development of depressive-like behavior following peripheral nerve injury. Mol. Psychiatry.

[32] Zhang Y.P., Wang H.Y., Zhang C., Liu B.P., Peng Z.L., Li Y.Y., Liu F.M., Song C. (2018). Mifepristone attenuates depression-like changes induced by chronic central administration of interleukin-1beta in rats. Behav. Brain Res..

[33] Yoshimura R., Kishi T., Atake K., Katsuki A., Iwata N. (2018). Serum Brain-Derived Neurotrophic Factor, and Plasma Catecholamine Metabolites in People with Major Depression: Preliminary Cross-Sectional Study. Front. Psychiatry.

[34] Serra M.P., Poddighe L., Boi M., Sanna F., Piludu M.A., Corda M.G., Giorgi O., Quartu M. (2017). Expression of BDNF and trkB in the hippocampus of a rat genetic model of vulnerability (Roman low-avoidance) and resistance (Roman high-avoidance) to stress-induced depression. Brain Behav..

[35] Zhu S., Shi R., Wang J., Wang J.F., Li X.M. (2014). Unpredictable chronic mild stress not chronic restraint stress induces depressive behaviours in mice. Neuroreport.

[36] Reisinger S., Khan D., Kong E., Berger A., Pollak A., Pollak D.D. (2015). The poly(I:C)-induced maternal immune activation model in preclinical neuropsychiatric drug discovery. Pharmacol. Ther..

[37] Levy-Gigi E., Szabo C., Kelemen O., Keri S. (2013). Association among clinical response, hippocampal volume, and FKBP5 gene expression in individuals with posttraumatic stress disorder receiving cognitive behavioral therapy. Biol. Psychiatry.

[38] Szabo C., Kelemen O., Keri S. (2014). Changes in FKBP5 expression and memory functions during cognitive-behavioral therapy in posttraumatic stress disorder: A preliminary study. Neurosci. Lett..

[39] Chen J., Wang Z.Z., Zuo W., Zhang S., Chu S.F., Chen N.H. (2016). Effects of chronic mild stress on behavioral and neurobiological parameters—Role of glucocorticoid. Horm. Behav..

[40] Meyer U. (2014). Prenatal poly(i:C) exposure and other developmental immune activation models in rodent systems. Biol. Psychiatry.

[41] Cabral G.A., Griffin-Thomas L. (2009). Emerging role of the cannabinoid receptor CB2 in immune regulation: Therapeutic prospects for neuroinflammation. Expert Rev. Mol. Med..

[42] Palazuelos J., Aguado T., Egia A., Mechoulam R., Guzman M., Galve-Roperh I. (2006). Non-psychoactive CB2 cannabinoid agonists stimulate neural progenitor proliferation. FASEB J..

[43] Hollins S.L., Zavitsanou K., Walker F.R., Cairns M.J. (2016). Alteration of transcriptional networks in the entorhinal cortex after maternal immune activation and adolescent cannabinoid exposure. Brain Behav. Immun..

[44] Chadwick B., Miller M.L., Hurd Y.L. (2013). Cannabis Use during Adolescent Development: Susceptibility to Psychiatric Illness. Front. Psychiatry.

[45] Gomes F.V., Rincon-Cortes M., Grace A.A. (2016). Adolescence as a period of vulnerability and intervention in schizophrenia: Insights from the MAM model. Neurosci. Biobehav. Rev..

[46] Ratajczak P., Wozniak A., Nowakowska E. (2013). Animal models of schizophrenia: Developmental preparation in rats. Acta Neurobiol. Exp..

[47] Bailey K.A., Baker A.L., McElduff P., Kavanagh D.J. (2016). The Influence of Parental Emotional Neglect on Assault Victims Seeking Treatment for Depressed Mood and Alcohol Misuse: A Pilot Study. J. Clin. Med..

[48] Wang Q., Dong X., Wang Y., Liu M., Sun A., Li N., Lin Y., Geng Z., Jin Y., Li X. (2017). Adolescent escitalopram prevents the effects of maternal separation on depression- and anxiety-like behaviours and regulates the levels of inflammatory cytokines in adult male mice. Int. J. Dev. Neurosci..

[49] Malkesman O., Lavi-Avnon Y., Maayan R., Weizman A. (2008). A cross-fostering study in a genetic animal model of depression: Maternal behavior and depression-like symptoms. Pharmacol. Biochem. Behav..

[50] Llorente R., Llorente-Berzal A., Petrosino S., Marco E.M., Guaza C., Prada C., Lopez-Gallardo M., Di Marzo V., Viveros M.P. (2008). Gender-dependent cellular and biochemical effects of maternal deprivation on the hippocampus of neonatal rats: A possible role for the endocannabinoid system. Dev. Neurobiol..

[51] Lopez-Gallardo M., Llorente R., Llorente-Berzal A., Marco E.M., Prada C., Di Marzo V., Viveros M.P. (2008). Neuronal and glial alterations in the cerebellar cortex of maternally deprived rats: Gender differences and modulatory effects of two inhibitors of endocannabinoid inactivation. Dev. Neurobiol..

[52] Recamier-Carballo S., Estrada-Camarena E., Lopez-Rubalcava C. (2017). Maternal separation induces long-term effects on monoamines and brain-derived neurotrophic factor levels on the frontal cortex, amygdala, and hippocampus: Differential effects after a stress challenge. Behav. Pharmacol..

[53] Van Bodegom M., Homberg J.R., Henckens M. (2017). Modulation of the Hypothalamic-Pituitary-Adrenal Axis by Early Life Stress Exposure. Front. Cell. Neurosci..

[54] Page G.G., Corwin E.J., Dorsey S.G., Redeker N.S., McCloskey D.J., Austin J.K., Guthrie B.J., Moore S.M., Barton D., Kim M.T. (2018). Biomarkers as Common Data Elements for Symptom and Self-Management Science. J. Nurs. Scholarsh..

[55] Buckley N.E. (2008). The peripheral cannabinoid receptor knockout mice: An update. Br. J. Pharmacol..

[56] Commons K.G., Cholanians A.B., Babb J.A., Ehlinger D.G. (2017). The Rodent Forced Swim Test Measures Stress-Coping Strategy, Not Depression-like Behavior. ACS Chem. Neurosci..

[57] Maejima Y., Rita R.S., Santoso P., Aoyama M., Hiraoka Y., Nishimori K., Gantulga D., Shimomura K., Yada T. (2015). Nasal oxytocin administration reduces food intake without affecting locomotor activity and glycemia with c-Fos induction in limited brain areas. Neuroendocrinology.

